# miRNA-148a serves as a prognostic factor and suppresses migration and invasion through Wnt1 in non-small cell lung cancer

**DOI:** 10.1371/journal.pone.0171751

**Published:** 2017-02-15

**Authors:** Yong Chen, Lingfeng Min, Chuanli Ren, Xingxiang Xu, Jianqi Yang, Xinchen Sun, Tao Wang, Fang Wang, Changjiang Sun, Xizhi Zhang

**Affiliations:** 1 Department of Medical Oncology, Northern Jiangsu People's Hospital, Clinical Medical College of Yangzhou University, Yangzhou, Jiangsu, China; 2 Departments of Respiratory Medicine, Northern Jiangsu People's Hospital, Clinical Medical College of Yangzhou University, Yangzhou, Jiangsu, China; 3 Departments of Clinical Medical Testing Laboratory, Northern Jiangsu People's Hospital, Clinical Medical College of Yangzhou University, Yangzhou, Jiangsu, China; 4 Department of Radiotherapy, the First Affiliated Hospital of Nanjing Medical University, Nanjing, Jiangsu, China; Beijing Cancer Hospital, CHINA

## Abstract

Lung cancer is the leading cause of cancer death in the world, and aberrant expression of miRNA is a common feature during the cancer initiation and development. Our previous study showed that levels of miRNA-148a assessed by quantitative real-time polymerase chain reaction (qRT-PCR) were a good prognosis factor for non-small cell lung cancer (NSCLC) patients. In this study, we used high-throughput formalin-fixed and paraffin-embedded (FFPE) lung cancer tissue arrays and in situ hybridization (ISH) to determine the clinical significances of miRNA-148a and aimed to find novel target of miRNA-148a in lung cancer. Our results showed that there were 86 of 159 patients with low miRNA-148a expression and miRNA-148a was significantly down-regulated in primary cancer tissues when compared with their adjacent normal lung tissues. Low expression of miRNA-148a was strongly associated with high tumor grade, lymph node (LN) metastasis and a higher risk of tumor-related death in NSCLC. Lentivirus mediated overexpression of miRNA-148a inhibited migration and invasion of A549 and H1299 lung cancer cells. Furthermore, we validated Wnt1 as a direct target of miRNA-148a. Our data showed that the Wnt1 expression was negatively correlated with the expression of miRNA-148a in both primary cancer tissues and their corresponding adjacent normal lung tissues. In addition, overexpression of miRNA-148a inhibited Wnt1 protein expression in cancer cells. And knocking down of Wnt-1 by siRNA had the similar effect of miRNA-148a overexpression on cell migration and invasion in lung cancer cells. In conclusion, our results suggest that miRNA-148a inhibited cell migration and invasion through targeting Wnt1 and this might provide a new insight into the molecular mechanisms of lung cancer metastasis.

## Introduction

Lung cancer is a commonly diagnosed and highly aggressive tumor worldwide. In china, both incidence and mortality rates of lung cancer are the highest, and there are no declining trends from 2000 to 2011[1]. In 2015, it is predicted that there will be about 733,000 newly diagnosed lung cancer cases and about 610,000 Chinese will die from lung cancer. Lung cancer carcinogenesis is a multistep process through accumulation of genetic and epigenetic alterations[2–3]. But the molecular mechanisms of pathogenesis and cancer progression are not well understood. Thus, further elucidation of possible targets for preventing the initiation and progression of lung cancer is imperative and essential for the development of effective therapy.

MicroRNAs (miRNAs) are small endogenously expressed non-coding RNA molecules that bind to imperfect sequence homology sites of mRNA's 3' untranslated regions (3'UTR), which causes either degradation or inhibition of putative protein translation[4]. Recently, miRNA-148a has been demonstrated to have important roles in cancer initiation and progression. Under normal physiological conditions, the miRNA-148a gene is expressed in various human tissues[5–7]. Specific over-expressed miRNA-148a may be involved in maintaining tissue homeostasis, and the low expression levels of miRNA-148a might induce a neoplasia[8]. Currently, the down-regulated expression of miRNA-148a can be detected in various cancers including non-small cell lung cancer (NSCLC) [9–10]. It has been shown to suppress tumor proliferation, epithelial-mesenchymal transition (EMT), cancer stem cells-like properties, immune evasion, angiogenesis, invasion and drug-resistant and promote apoptosis in different types of human tumors[10]. Furthermore, miRNA-148a can also serve as a effectively predictive biomarker of clinicopathological significance and prognosis in a variety of cancers[11–15]. In NSCLC, our previous study found that the expression of miRNA‑148a assessed by quantitative real-time polymerase chain reaction (qRT-PCR) was closely associated with patients’ clinicopathological characteristics and survival[16]. Thus, a better understanding of the changes in miRNA-148a expression may lead to a better understanding of NSCLC development, as well as to possible improvements in the diagnosis and treatment of NSCLC.

In the present study, for the first time, we used in situ hybridization(ISH) and high-throughput formalin-fixed and paraffin-embedded (FFPE) lung cancer tissue arrays to determine miRNA‑148a expression files and their relationships with clinicopathological characteristics and survival in NSCLC patients. We further analyzed the effects of miRNA-148a on migration and invasion of NSCLC cells in vitro. Importantly, we used dual-luciferase reporter assay to validate miRNA-148a’s potential target wingless-type protein 1 (Wnt1). These findings will provide new insights into the molecular mechanisms of metastasis and provide a therapeutic strategy for the treatment of lung cancer.

## Materials and methods

### Ethical approval of the study protocol

The present study was approved by the Ethics Committee of Northern People's Hospital (Jiangsu, China).

### Tissue arrays

FFPE lung cancer tissue arrays were obtained from the National Engineering Center for BioChips in Shanghai, China. Tissue arrays consisted of 165 pairs of human primary NSCLC (90 adenocarcinomas and 75 squamous cell carcinomas) and their adjacent normal lung tissues. For squamous cell carcinoma patients, the operation times were between July 2004 and November 2007, and the follow-up data was July 2012. For adenocarcinoma patients, the operation times were between July 2004 and June 2009, and the follow-up data was August 2014. The patients' clinical records and histopathological diagnoses were fully documented. Survival times was calculated in months and defined as the time from operation time until death, or censored if no death was noted at follow-up date. Similar to previous reports[17–20], there were 8 (4.85%) patients who died within 3 month after operation, and the cause of this early mortality after surgical resection was closely related to postoperative complications with no sign of recurrent disease. So these patients were also defined as censored data.

### miRNA ISH analysis and scoring

ISH was carried out with miRCURY locked nucleic acid(LNA) detection probe for miRNA-148a(Exiqon, 38050–15, probe concentration 25 μM, Vedbaek, Denmark) to detect expression levels of human miRNA-148a in FFPE tissue arrays as previously described[21] with slight modifications. A final concentration of 500 nmol/L of digoxigenin double-labeled LNA-modified probe was added to the hybridization solution and hybridized according to the manufacturer's instructions. After the incubation of slides with 4-nitroblue-tetrazolium and incubation with nuclear fast red (Roche Applied Science, Indianapolis, IN, USA), the sample images were captured with 200× magnification. ISH signals for miRNA-148a were scored by combining the staining intensity and the proportion of positive cells, as previously described [22]. The intensity of staining cells was scored as: negative (0), weak (1), intermediate (2), and strong (3). Another score was given for the proportion of positive cells was as follows: 0 points: none positive cells, 1 point: 1 to 24% of positive cells, 2 points: 25 to 49% of positive cells, 3 points: 50 to 74% of positive cells, 4 points: 75 to 100% of positive cells. The staining index (SI) was calculated by multiplying the staining intensity and the proportion of positive cells. A final SI score was taken to define expression levels as previously described[23], and low expression of miRNA-148a was described as SI score <6, whereas high expression of miRNA-148a was described as SI score ≥6.

### Cell culture

Human NSCLC cell line A549 and H1299 was purchased from the Cell Resource Center, Shanghai Institute of Biochemistry and maintained in RPMI-1640 (Hyclone, GE Healthcare Life Sciences, Piscataway, NJ, USA) with 10% fetal bovine serum (Wisent, Quebec, Canada) at 37℃ in a humidified atmosphere containing 5% carbon dioxidein.

### Establishment of stably expressing miRNA-148a lung cancer cell lines by lentivirus

A549 and H1299 cells with stable miRNA-148a expression and their parental cell lines were established using the lentiviral expression system. Briefly, Lentiviruses containing miRNA-148a (LV-148a) or negative control (LV-NC) were constructed by the GeneChem Company (Shanghai, China). All the lentiviral vectors expressed enhanced green fluorescent protein (GFP) and puromycin resistance gene, which allowed for measuring the infection efficiency and selecting the specific cells of interest. Cells were infected with LV-NC or LV-148a vectors, followed by selection for stable lines using 5 μg/ml of puromycin (Clontech, Palo Alto, CA, USA). The resulting stable lines were used for further analysis.

### RNA isolation and expression analysis of miRNA-148a by qRT-PCR

Total RNA was extracted using TRIzol reagent (Invitrogen Life Technologies, Carlsbad, CA, USA) according to the manufacturer's instructions. At last, the concentration of RNA was measured by using a NanoDrop-1000 (Thermo Fisher Scientific, Waltham, MA, USA). cDNA synthesis was then performed according to the manufacturer's recommendation. Briefly, RNA was reverse transcribed (RT) using a ReverAid First Strand cDNA kit (Thermo Fisher Scientific) in combination with a stem-loop primer for miRNA-148a. U6 snRNA was used as an internal control for normalizing the miRNA-148a expression levels. After the RT reaction, expressions of miRNA were detected using LightCycler 480 SYBR-Green I Master and the LightCycler 480 Real-Time PCR system (both from Roche Applied Science). The relative miRNA-148a expression was calculated by the 2^*-ΔCt*^ method. All primers for miRNA-148a and U6 were designed as previously reported [24] and synthesized by Shenggong Biotech Co., Ltd. (Shanghai, China).

### Wound-healing assay

The stably expressing miRNA-148a cells (1×10^6^ per well) were trypsinized and seeded in 24-well plates, cultured overnight in complete medium. The monolayers of cells were scratched using a 200-μl pipette tip. Cells were then washed with culture medium to remove cellular debris and allowed to culture again up to 24 h in serum-free medium. Images were captured under an Eclipse Ti-U (Nikon, Kanagawa, Japan) inverted microscope following wounding (0 and 24h). The relative surface traveled by the leading edge was assessed using Image-Pro Plus version 6.0 software.

### Transwell migration assays

Stably transfected cells (2.5×10^4^ cells) in serum-free medium were planted to the upper transwell chamber (Corning Incorporated, Corning, NY, USA, 24-well insert, pore size: 8 mm) of the insert pre-coated with Matrigel (BD Biosciences, Bedford, MA, USA). Complete medium (500 μl) containing 10% FBS was placed to the lower chambers as a chemoattractant. After 40 h of incubation, the cells that did not migrate or invade through the pores were carefully wiped off by wet cotton swab. The inserts were then stained with 20% methanol and 0.2% crystal violet, imaged with an Eclipse Ti-U inverted microscope, and invaded cell number per field was counted by Image-Pro Plus version 6.0.

### Western blot analysis

Total proteins from cells were homogenized using RIPA lysis buffer. And the protein concentrations were determined by BCA protein reagent (Pierce Chemical Co., Rockford, IL, USA). Western blot was performed as described previously[25]. Primary antibodies were as follows: rabbit polyclonal anti-wnt1 antibody (1:500; Abcam ab15251, Cambridge, UK)), mouse monoclonal β-actin (1:1000; Santa Cruz, Santa Cruz, CA, USA), horseradish peroxidase (HRP) -conjugated anti-rabbit secondary antibody (1: 5000; Santa Cruz Biotechnology), and anti-mouse secondary antibody (1:5000; Santa Cruz Biotechnology). The anti-β-actin antibody was used as the internal reference. Finally, the active bands were visualized with enhanced chemiluminescence system.

### Dual-luciferase reporter assay

All the vectors in luciferase-reporter assay system were purchased from GeneChem (Shanghai, China). Briefly, double-stranded oligonucleotides corresponding to the wild- or mutant-type 3’-UTR of Wnt1 and were synthesized and subcloned into the GV272 plasmid reporter vector (SV40-Luciferase-MCS). The miRNA-148a gene was cloned into the GV268 plasmid vector (CMV-MCS-SV40-Neomycin). The scrambled oligonucleotides (NC) were cloned into the GV272 and GV268 vectors and served as control groups respectively. 293T cells (1×10^5^) were co-transfected with luciferase reporter plasmids and miRNA-148a plasmids, using lipofectamine 2000 (Invitrogen, Carlsbad, CA, USA). Twenty four hours after transfection, luciferase assays were performed using the dual-luciferase reporter assay system (Promega, USA). Normalized luciferase activity was reported as luciferase activity/Renilla luciferase activity.

### Oligonucleotide construction and transient transfection

The siRNA targeting human Wnt1 and non-targeting siRNA were purchased from GenePharma Co., Ltd (Shanghai). Transfections were performed using Lipofectamine RNAiMAX (Invitrogen) according to the manufacturer’s instructions. For the RNA interference study, we tested three siRNAs designed using the target gene sequences and selected the siRNA that resulted in the greatest inhibition of the target protein.

### Immunohistochemical staining

Immunohistochemical staining was carried out with immunohistochemistry kit (Zhong Shan-Golden Bridge Biological Technology, Beijing, China) according to the manufacturer’s protocol. A rabbit polyclonal antibody for Wnt1 (1: 1000; Abcam) was used as primary antibody. The expression of Wnt1 was evaluated by the scoring system combining staining intensity and proportion of positive cells[26]. Quantification of the staining intensity of Wnt1 was carried out through image analysis in the same manner as ISH.

### Statistical analysis

Statistical analysis was performed using SPSS 13.0 statistical software. Data are shown as means ± standard deviation (SD), and one-way analysis of variance (ANOVA) was used to compare the differences among groups. Nonparametric test was used to compare the difference of miRNA-148a expression between primary NSCLC and their adjacent normal lung tissues. The Chi-square test was used to examine the associations between miRNA-148a expression and various clinicopathologic characteristics. Univariate Kaplan-Meier method was performed to calculate overall survival curves according to miRNA-148a expression. Survival differences according to expression were analyzed using the log-rank test. Multivariable Cox proportional hazards analysis were performed to determine independent prognostic factors of patient survivals. The Spearman correlation analysis was carried out to analyze the association between the expression of miRNA-148a and expression of its potential target Wnt1 proteins. A difference of *P*<0.05 between groups was considered as a statistically significant result.

## Results

### Lack of miRNA-148a expression in NSCLC is associated with tumor progression and poor clinical outcome

**Fig 1:**
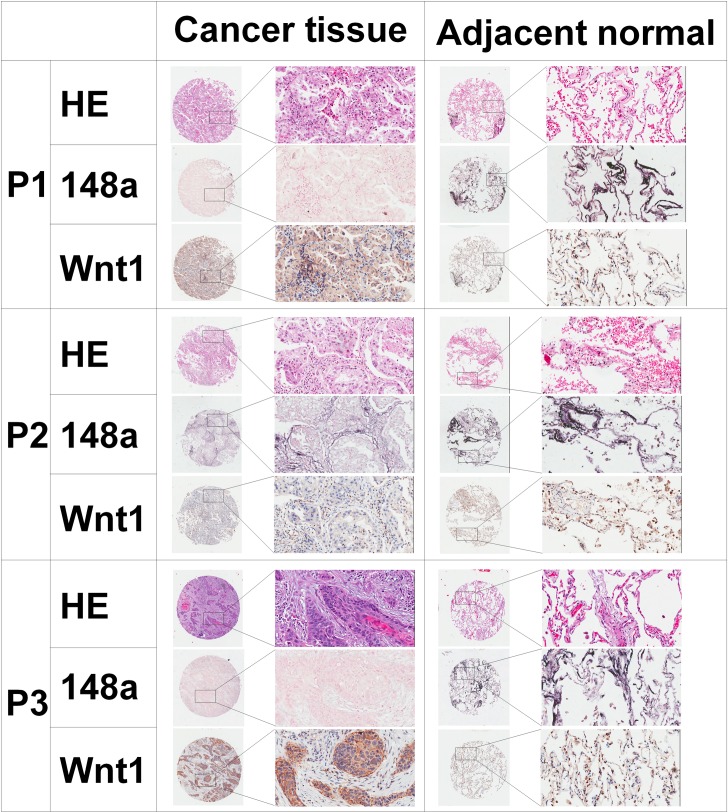
HE, ISH and IHC analysis of NSCLC tissue and corresponding adjacent normal lung tissues from 3 representative patients (P1 and P2: adenocarcinoma; P3: squamous cell carcinoma). HE, ISH for miRNA-148a and IHC for Wnt1 from the same tissue area are shown. ISH shows differential expressions of miRNA-148a in primary tissues compared to adjacent normal lung tissues. Weak miRNA-148a staining in cancer tissues with corresponding strong miRNA-148a staining in adjacent normal lung tissues (P1 and P3). Negative correlation between miRNA-148a and Wnt1 expression in NSCLC samples. Increased Wnt1 immunoreactivity in cancer tissues with low levels of miRNA-148a assessed by ISH (P1 and P3). Decreased Wnt1 immunoreactivity in lung cancer tissues with high level of miRNA-148a (P2). Increased miRNA-148a staining in adjacent normal lung tissues with low levels of Wnt1 staining (P1, P2 and P3). HE: hematoxylin-eosin staining, ISH: in situ hybridization and IHC: immunohistochemistry.

**Fig 2:**
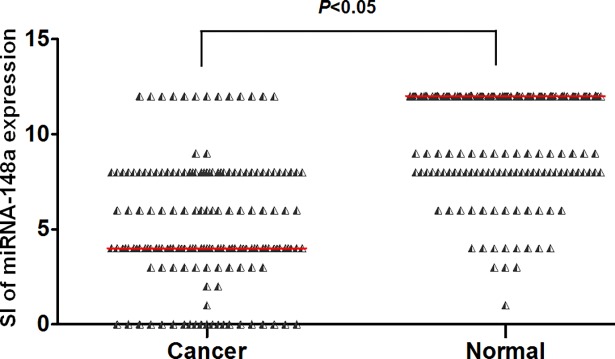
The staining index (SI) of miRNA-148a expressions in FFPE primary lung cancer tissues and their adjacent normal lung tissues. The expression of miRNA-148a in cancer tissues was significantly lower than that in adjacent normal tissues (*P*<0.05) and the median SI of miRNA-148a expressions between the two groups are marked with a red line in the graph.

To further evaluate miRNA-148a expressions in NSCLC, we sought to examine a large cohort of clinical NSCLC samples. Tissue arrays comprising 165 pairs of FFPE primary NSCLC and their adjacent normal lung tissues were examined for miRNA-148a expression by using ISH. All patients were followed up to 5 years or more. The clinical and pathologic characteristics of patients were summarized in Table 1. The median age at diagnosis of the study population was 63.50 years (range, 30 to 84 years). A total of 159 primary cancer tissues and 164 adjacent normal lung tissues were successfully detected in tissue arrays. There were 86 patients with low expressed miRNA-148a in FFPE primary cancer tissues. Fig 1 showed representative images of the ISH results. MiRNA-148a was found to be down-regulated in cancer tissues compared with adjacent normal lung tissues (Fig 2, *P*<0.05).

**Table 1:** Clinical and pathologic characteristics of the NSCLC patients.

		Frequency			Frequency
Age(years)	≤60	62	LN^c^ metastasis	Negative	79
	>60	102		Positive	78
	Unknown	1		Unknown	8
Gender	Male	119	Tumor size(cm)	≤3	55
	Female	46		3~7	97
Histologic type	SCC^a^	75		>7	11
	Ad^b^	90		Unknown	2
Tumor grade	Ⅰ	9	T stage	1	34
	Ⅱ	118		2	89
	Ⅲ	38		3	41
				Unknown	1

^a^Squamous cell carcinoma

^b^Adenocarcinoma

^c^Lymph node

Levels of miRNA-148a expression were further analyzed to determine its relationship with the clinicopathologic characteristics of NSCLC. Among the successfully detected cases, patients were categorized into two groups of high or low expression. As shown in Table 2, miRNA-148a expression strongly correlated with the tumor grade and lymph node (LN) metastasis (*P* = 0.008 and 0.018 respectively), whereas no difference was seen among age (*P* = 0.674), gender (*P* = 0.776), histologic type (*P* = 0.100), tumor size (*P* = 0.654) and T stage (*P* = 0.819). This result supports that low level of miRNA-148a expression is associated with NSCLC clinical progressions.

**Table 2:** Correlation between miRNA-148a expression and multiple clinicopathological characteristics in NSCLC patients.

		miRNA-148a expression		Total	*x*^2^	*P* value
		Low	High			
Age(year)	≤60	31	29	60	0.177	0.674
	>60	54	44	98		
	Total	85	73	158		
Gender	Male	63	52	115	0.081	0.776
	Female	23	21	44		
	Total	86	73	159		
Histologic type	SCC^a^	43	27	70	2.714	0.100
	Ad^b^	43	46	89		
	Total	86	73	159		
Tumor grade	Ⅰ~Ⅱ	59	63	122	6.925	0.008
	Ⅲ	27	10	37		
	Total	86	73	159		
LN^c^ metastasis	Negative	34	42	76	5.645	0.018
	Positive	48	27	75		
	Total	82	69	151		
Tumor size(cm)	≤3	25	26	51	0.849	0.654
	3~7	54	42	96		
	>7	6	4	10		
	Total	85	72	157		
T stage	T1	15	15	30	0.400	0.819
	T2	47	41	88		
	T3	23	17	40		
	Total	85	73	158		

^a^Squamous cell carcinoma

^b^Adenocarcinoma

^c^Lymph node

**Fig 3:**
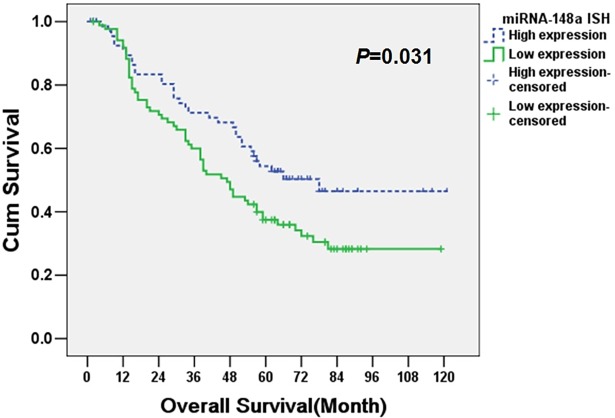
Kaplan-Meier curves for overall survival of NSCLC patients according to miRNA-148a expressions. MiRNA-148a down-regulation significantly correlated with shorter overall survivals.

To further evaluate effects of miRNA-148a expression on the survival in NSCLC patients, a log-rank test, kaplan-meier survival and cox proportional hazards analysis were performed. The miRNA-148a expression level in NSCLC displayed a significant correlation with the patients’ overall survival time (*P* = 0.031). Specifically, the estimate median survival time in patients with tumors expressed low levels of miRNA-148a was only 47.0 months, whereas the estimate median survival time was 78.0 months in patients whose tumors expressed high levels of miRNA-148a. As shown in Fig 3, the cumulative 5-year survival rate was 54.4% in the high miRNA-148a expression group, whereas it was only 37.4% in the low expression group. Finally, multivariable cox proportional hazards analysis were performed to determine whether the miRNA-148a expression level was an independent prognostic factor of patient survivals. As shown in Table 3, miRNA-148a expression, histologic type and LN metastasis were identified as independent prognostic factors for NSCLC. Patients with low expression of miRNA-148a had a significantly higher risk of tumor-related death (hazard ratio, 1.594; 95% CI, 1.008–2.521, *P* = 0.046) compared with those expressing high levels of miRNA-148a.

**Table 3:** Multivariable Cox regression analysis of clinicopathological variables and cumulative survival of patients with NSCLC.

		n	*P* values	Relative risk	95% confidence interval	
					Lower	Upper
Age(years)	≤60	58		1.000		
	>60	92	0.175	1.374	0.868	2.175
Gender	Male	108		1.000		
	Female	42	0.831	1.056	0.640	1.744
Histologic type	SCC^a^	64		1.000		
	Ad^b^	86	0.001	2.376	1.400	4.032
miRNA-148a expression	High	69		1.000		
	Low	81	0.046	1.594	1.008	2.521
LN^c^ metastasis	Negative	75		1.000		
	Positive	75	0.002	2.055	1.290	3.273
Tumor size(cm)			0.233			
	≤3	48		1.000		
	3~7	92	0.135	1.472	0.886	2.444
	>7	10	0.173	1.900	0.754	4.784

^a^Squamous cell carcinoma

^b^Adenocarcinoma

^c^Lymph node

Taken together, our results suggest that miRNA-148a could be a valuable marker of NSCLC progression, and that low miRNA-148a expression is associated with poor overall survival in NSCLC patients.

### Over-expression of miRNA-148a suppresses cells migration and invasion of NSCLC cells in vitro

**Fig 4:**
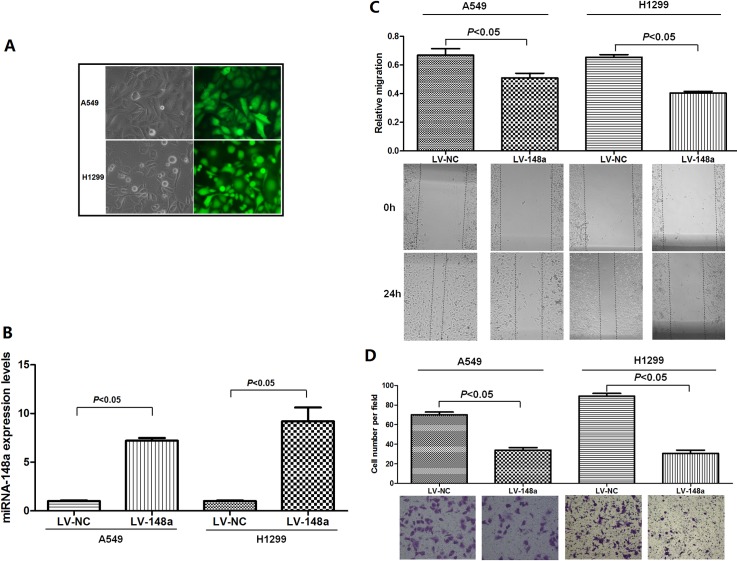
miRNA-148a suppresses cells migration and invasion of lung cancer A549 and H1299 cells in vitro. **(A)** Optical (left) and their corresponding fluorescence (right) images of A549 and H1299 cells stably infected with miRNA-148a-overexpression lentivirus vectors (LV-148a). Cells were infected with LV-148a and selected by 5 μg/ml of puromycin. (B) Expression of miRNA-148a in A549 and H1299 cells stably transfected with LV-148a or control vector (LV-NC) using qRT-PCR. (C) Stable transfection of A549 and H1299 cells with LV-148a resulted in inhibition of cell migration using wound-healing assay. (D) Transfection of A549 and H1299 cells with LV-148a inhibited cell invasion using transwell migration assays. Images were captured at 200× optical magnification.

Considering the correlation between miRNA-148a expressions and lung cancer progression, we further investigated the effect of miRNA-148a on the migration and invasion abilities in lung cancer cell line. A549 and H1299 cell lines were infected with either LV-148a or LV-NC, and then we established two stable miRNA-148a expression cell lines (Fig 4A). Compared with the expression of control group, qRT-PCR results showed that miRNA-148a was significantly up-regulated in both stably infected cell lines (Fig 4B).

Next, we performed wound-healing assay and transwell assay to determine the function of miRNA-148a in lung cancer cell lines. The migration and invasion abilities of stable miRNA-148a expression cells were significantly inhibited when compared with control cells (Fig 4C and 4D). Therefore, these data further implied the biological importance of miRNA-148a in lung cancer progression.

### miRNA-148a regulates the expression of Wnt1 in NSCLC cell line

**Fig 5:**
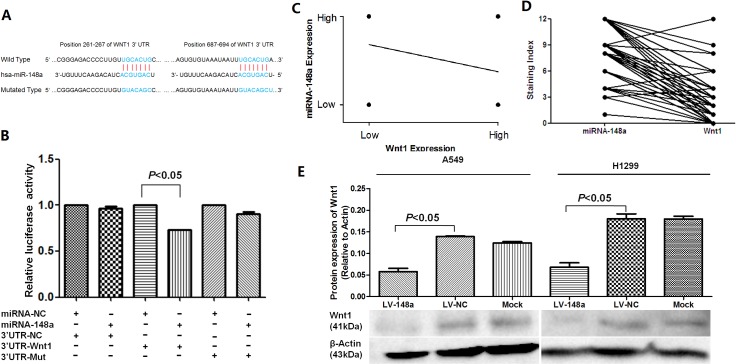
Experimental validation of Wnt1 as a direct target gene of miRNA-148a. (A) miRNA-148a potential binding sites and mutated sites on the 3’UTR of Wnt1. (B) 293T cells were co-transfected with plasmid vector containing either miRNA-148a or negative control (miRNA-NC) and luciferase reporter vector containing wild-type(3’UTR-Wnt1), mutant form(3’UTR-Mut) or negative control(3’UTR-NC) form of 3’UTR of Wnt1 and then assessed for luciferase reporter activity at 24 hours post-transfection. MiRNA-148a significantly reduced luciferase activities by directly bounding to Wnt1 3′-UTR. (C) Correlation analysis showed that the miRNA-148a expression was negatively related to the expression of Wnt-1 in NSCLC tissues (Coefficient = -0.276, *P* = 0.0004). (D) Association of miRNA-148a and Wnt1 staining scores in the same adjacent non-tumor lung tissues. (E) Protein expression of Wnt1 was reduced in A549 and H1299 cells with stable expression of miRNA-148a. β-actin was used as a loading control.

Recently, it has been reported that miRNA-148a down-regulated Wnt1 expression by directly targeting its 3’UTR in breast cancer and mesenchymal stem cells [13,27]. Using standard online software (TargetScan, http://www.targetscan.org), we found that there were two predicted binding sites in Wnt1 3’ UTR (Fig 5A). We cloned the Wnt1 wild-type and mutated-type 3′UTR into a luciferase reporter vector. Luciferase assay revealed that miRNA-148a significantly reduced luciferase activities by directly bounding to Wnt1 3′UTR (Fig 5B). However, mutation of the putative miRNA-148a binding sites in the Wnt1 3′UTR abrogated luciferase response to miRNA-148a. Next, we analyzed Wnt1 protein expressions using immunohistochemistry in the same tissue arrays which we used for miRNA-148a expression analysis by ISH (Fig 1). The expression of Wnt1 was negatively correlated with miRNA-148a in the spearman correlation analysis (Coefficient = -0.276, *P* = 0.0004) (Fig 5C). Meanwhile, in 161 adjacent normal tissues with a successful detection of both miRNA-148a and Wnt1, high staining scores of miRNA-148a were accompanied with low staining scores of Wnt1 in the same adjacent normal tissues (Fig 5D). We further performed western blot analysis to validate the effect of miRNA-148a on Wnt1 expression. As seen in Fig 5E, stable expression of miRNA-148a in A549 and H1299 cells inhibited Wnt1 protein expression. Taken together, these results indicated that Wnt1 was a direct target for miRNA-148a in NSCLC. The above results prompted us to determine whether miRNA-148a suppressed lung cancer metastasis through repressing Wnt1 expression.

### Inhibition of Wnt1 suppresses migration and invasion of NSCLC cells

**Fig 6:**
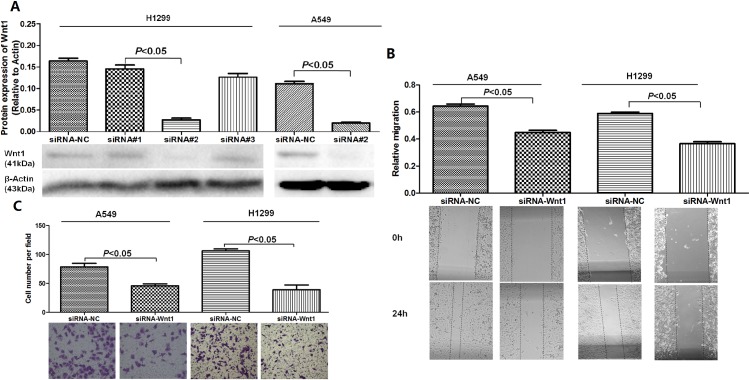
Inhibition of Wnt1 suppresses migration and invasion of A549 and H1299 cells. (A) Three siRNA were designed and Wnt1 protein level was detected by western blot to select the effective siRNA for the target protein. siRNA#2 exhibited greatest inhibition of Wnt1 protein and was further validated in A549 cells. β-actin was used as a loading control. (B) Both A549 and H1299 cells transfected with siRNA-Wnt1 resulted in inhibition of cell migration using wound-healing assay. (C) Transfection of A549 and H1299 cells with siRNA-Wnt1 inhibited cell invasion using transwell migration assays. Images were captured at 200× optical magnification.

To explore whether miRNA-148a suppresses metastasis of lung cancer cells through deregulating Wnt1, we studied the effects of silencing Wnt1 on the metastasis of lung cancer cells. Western blotting showed that 1 of 3 siRNAs targeting Wnt1 effectively inhibited Wnt1 expression in H1299 cells (Fig 6A). And this siRNA was further validated to inhibited Wnt1 expression in A549 cells. Using wound-healing assay and transwell assays, Wnt1 siRNA could inhibit the migration and invasion abilities of bothcancer cell lines (Fig 6B and 6C). These results were consistent with the effect of miRNA-148a overexpression, indicating that miRNA-148a regulates lung cells migration and invasion by directly targeting Wnt1.

## Discussion

In this study, we have showed that the frequent down-regulation of miRNA-148a in NSCLC tissue was significantly correlated with LN metastasis, tumor grade, and poor clinical outcome by using combining high-throughput tissue arrays and ISH. Our studies also revealed that miRNA-148a could suppress NSCLC cell migration and invasion in vitro. We also confirmed that Wnt1 was a direct and functional target of miRNA-148a using dual-luciferase reporter assay and its expression was correlated negatively with the expression of miRNA-148a. Further study showed that Wnt1 contributes to NSCLC cells migration and invasion abilities. Taken together, our results suggest that down-regulated miRNA-148a expression contributes to NSCLC progression by abrogating the inhibition of Wnt1 expression.

MiRNAs can be well preserved and easily recovered in extremely valuable FFPE tissues[28–29], which makes them a feasible alternative to fresh frozen tissue samples for miRNA expression analysis[30–32] and has great clinical significance for studies of miRNA as potential biomarkers for tumor diagnosis and predicting prognosis and response to the therapy[33]. However, a large proportion of previous studies were based on the RNA extraction from human FFPE cancer samples. Recently, many researchers have used ISH to determine cellular expression patterns of miRNA in FFPE tissues and their clinical significances [34–36]. In the present study, we detected miRNA-148a expression levels in FFPE tissue arrays using ISH. Our data demonstrated 159 cancer tissues and 164 adjacent normal lung tissues were successfully detected in FFPE NSCLC tissue arrays.

Aberrant expression of miRNA-148a has been reported in several cancers and miRNA-148a is widely considered to be a tumor suppressor in these cancers [10]. In lung cancer, it was also found to be down-regulated in primary tumor tissues or blood samples from patients [16,37–42]. But, to our knowledge, all studies, including our previous study [16], used qRT-PCR as the principal method in a limited cohort up to 73 cases[39]. And there is no other study except ours which has investigated the prognostic impact of miRNA-148a in NSCLC. Based on our previous study, we present a large-scale study combining high-throughput tissue arrays and ISH technology to further analyze the relationship between miRNA-148a expression and its clinical significances and outcomes again. Consistent with previous reports, we found a significantly decreased level of miRNA-148a in the primary cancer tissues than in the corresponding adjacent normal lung tissues. Intriguingly, all reports including our present study showed that low levels of miRNA-148a expression were significantly associated with LN metastasis [16,37–42]. In addition, our study also found that expression of miRNA-148a in primary tumor tissues was negatively associated with tumor grade. This finding indicated that miRNA-148a could play an important role in carcinogenesis and progression of tumor and may be useful to clinically predict metastasis in NCSLC patients.

In clinical, it is well accepted that an aggressive tumor behavior usually means a worse prognosis. In liver cancer, low expression of miRNA-148a significantly reduced the recurrence free survival rate and the overall survival [12]. And similar results could be further observed in gastric, colorectal and breast cancer [14–15,43]. In our present study, down-regulated miRNA-148a was also significantly associated with a poor survival. Furthermore, multivariable analysis also showed that miRNA-148a was an independent prognostic factor for overall survival. Our finding suggested that miRNA-148a might be useful to predict patients’ outcome.

To date, there are a lot of reports showing miRNA-148a could suppress metastatic behavior in many kinds of cancers including lung cancer [10]. Li et al found that miRNA-148a could suppressed NSCLC cell invasive and migratory abilities in vitro and suppressed cancer metastasis in vivo, while inhibition of miRNA-148a enhanced cell invasion and lung metastasis formation [39]. To further explore the role of miRNA-148a in tumor metastatic behavior in lung cancer, we established A549 and H1299 cell lines stably expressing miRNA-148a by lentivirus and used wound-healing and transwell migration assays to analyze. Together with results previously reported, our results suggest that miRNA-148a functions as a negative metastatic regulator for lung cancer.

There are many validated miRNA-148a’s target genes contributing to the tumor metastatic behavior, including MMP7[14], ROCK1 [42], SMAD2[44], Met[45] and cholecystokinin B receptor (CCK-BR)[46]. Furthermore, Shi et al recently reported that miRNA-148a could regulate adipogenesis by suppressing its target gene Wnt1 [27]. It was reported that the Wnt pathway played an important role in the regulation of several tumor behaviors. And Wnt1 is involved in regulating tumors cell migration and the epithelial-mesenchymal transition (EMT)[47–48]. Recently, in a meta-analysis, higher Wnt1 expression was also associated with poorer overall survival in lung cancer patients [49]. In the present study, we found that miRNA-148a inhibited Wnt1 protein expression in lung cancer cells and the expression of miRNA-148a was inversely related to the expression of Wnt1 in both lung cancer and their corresponding adjacent non-tumor lung tissues. Our results also showed that miRNA-148a could inhibit the migration and invasion of lung cancer cells by directly targeting Wnt1. Similarly, Jiang [13] and Yan [48] et al recently have reported that Wnt1 was a target gene of miRNA-148a in hepatocellular carcinoma and breast cancer cells. Taken together, our results suggest that miRNA-148a can suppress the migration and invasion of lung cancer cells by targeting Wnt1 and this will provide a new insight into the molecular mechanisms underlying cancer progression in NSCLC.

In conclusion, our studies suggest that miRNA-148a is significantly correlated with tumor progression and poor clinical outcome and can inhibit the migration and invasion of lung cancer cells by directly targeting Wnt1. Our results might provide a new strategy for lung cancer therapy. In future, the roles of Wnt signal regulatory network in miRNA-148a mediated regulation of invasion and migration should be further explored.

## Supporting information

S1 File Original, uncropped and unadjusted western blot images from which we made our figures in the manuscript. (DOCX)
