# Supplementary figures and images for: miRNA-148a serves as a prognostic factor and suppresses migration and invasion through Wnt1 in non-small cell lung cancer
Source: PLoS One. 2017 Feb 15;12(2):e0171751. doi: 10.1371/journal.pone.0171751 (PMC5310808; doi:10.1371/journal.pone.0171751)

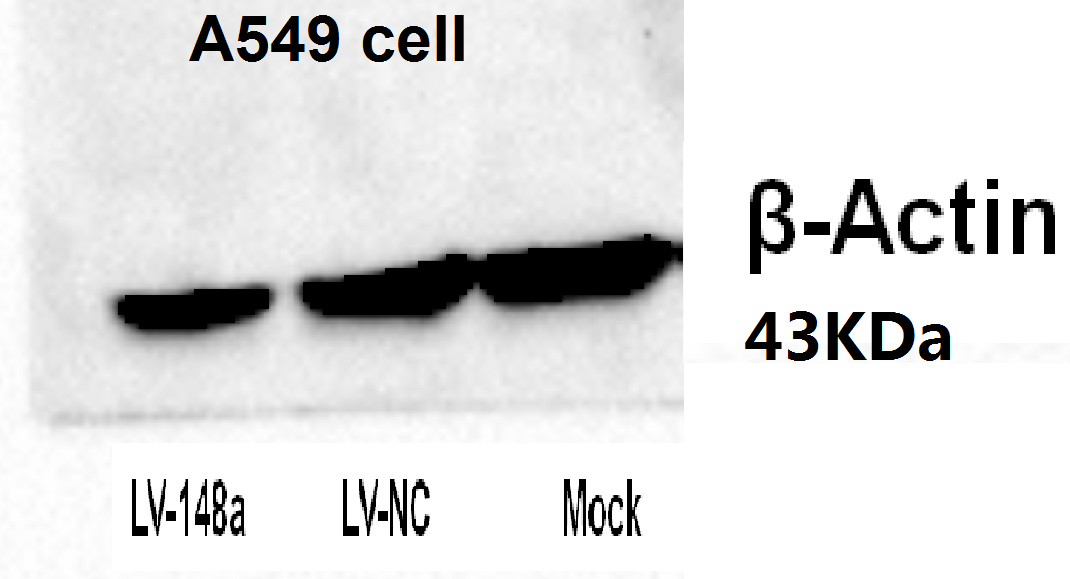

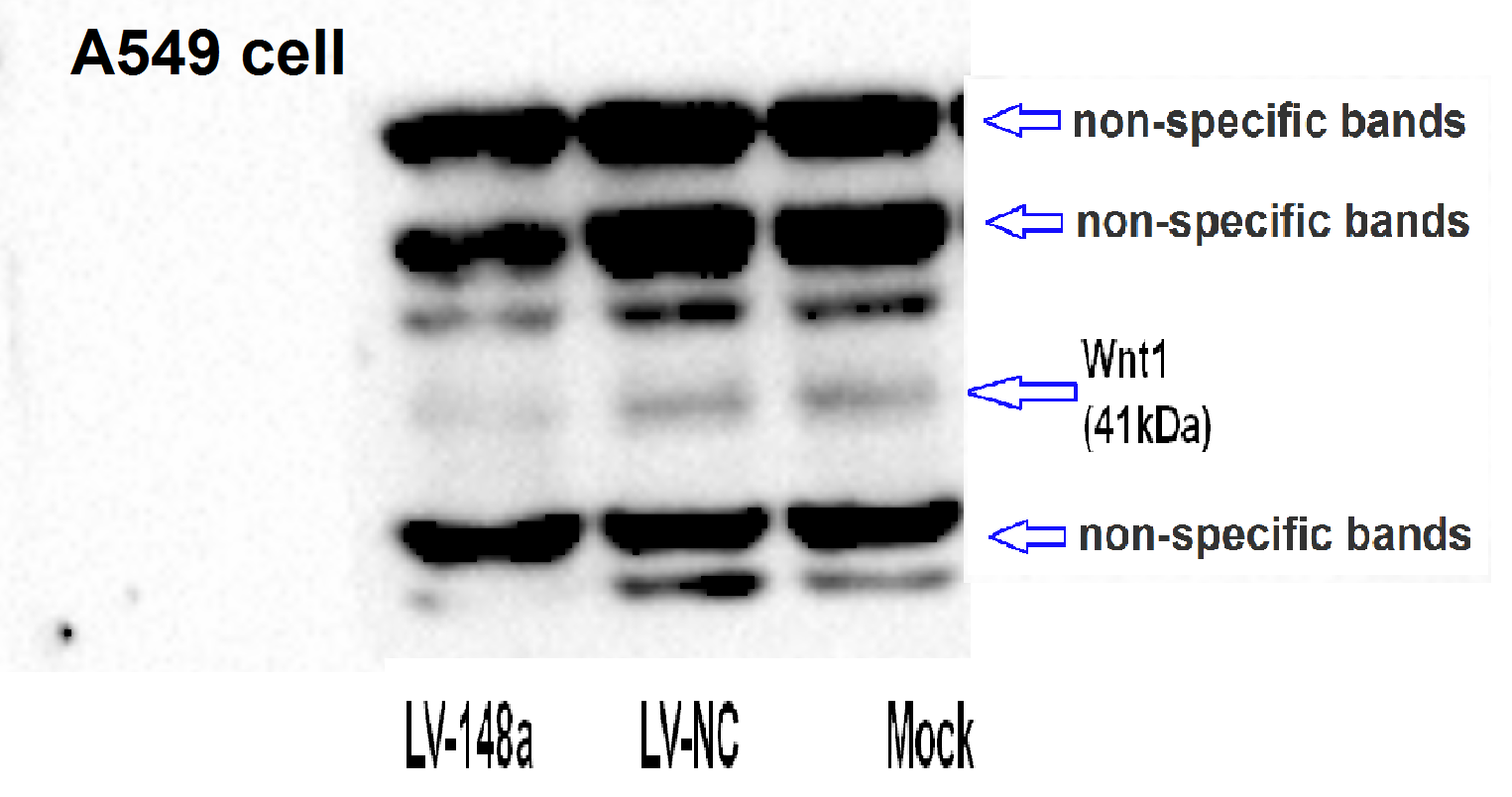

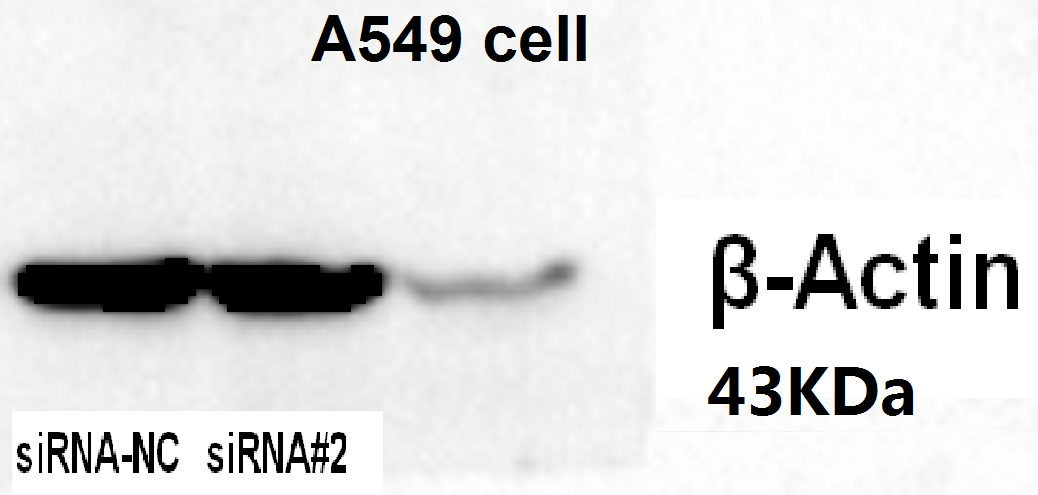

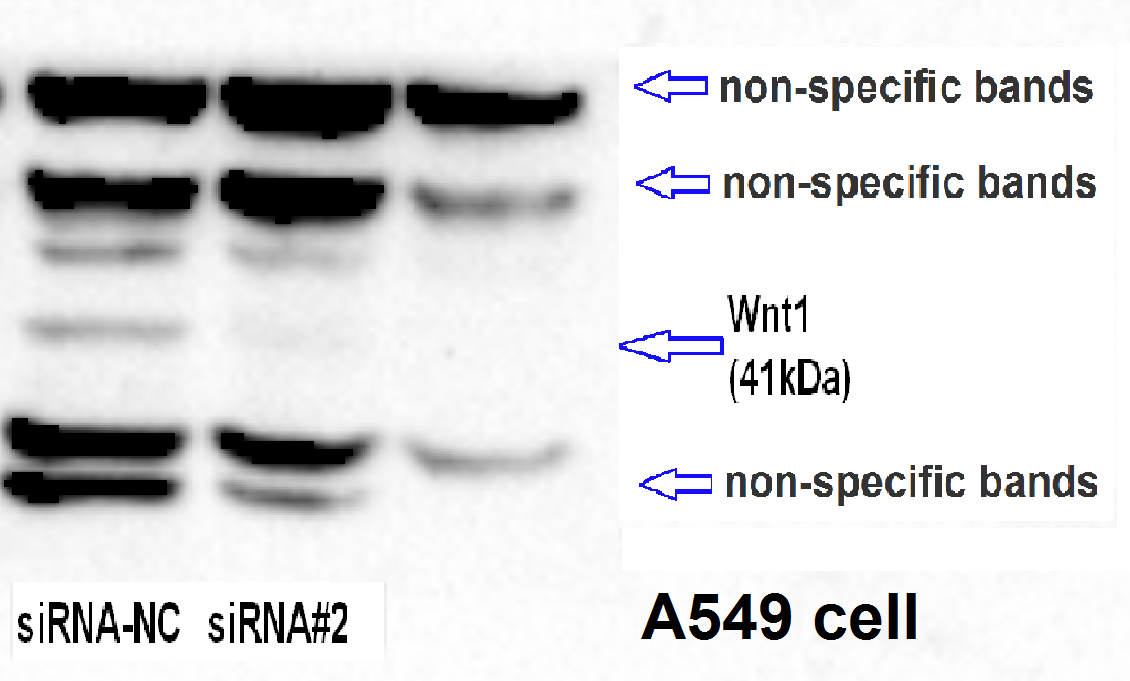

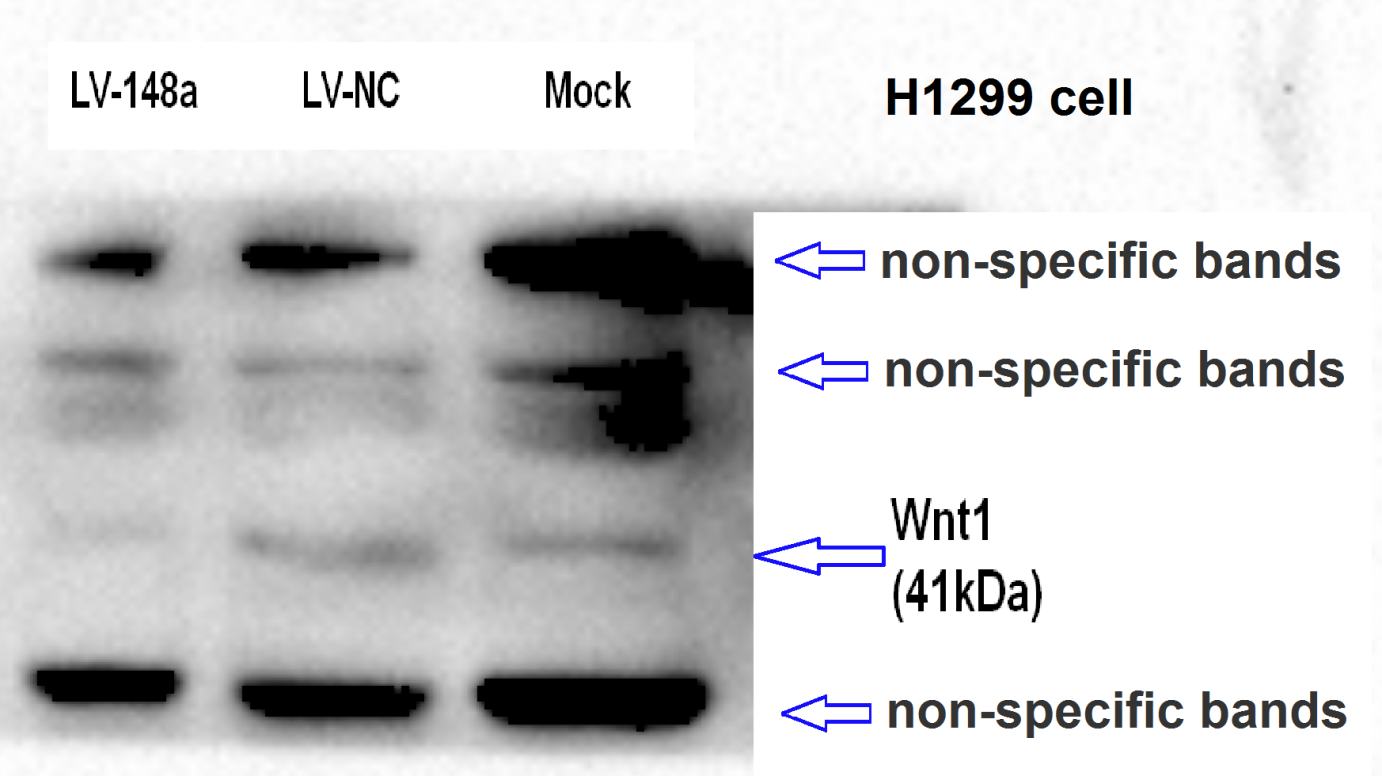

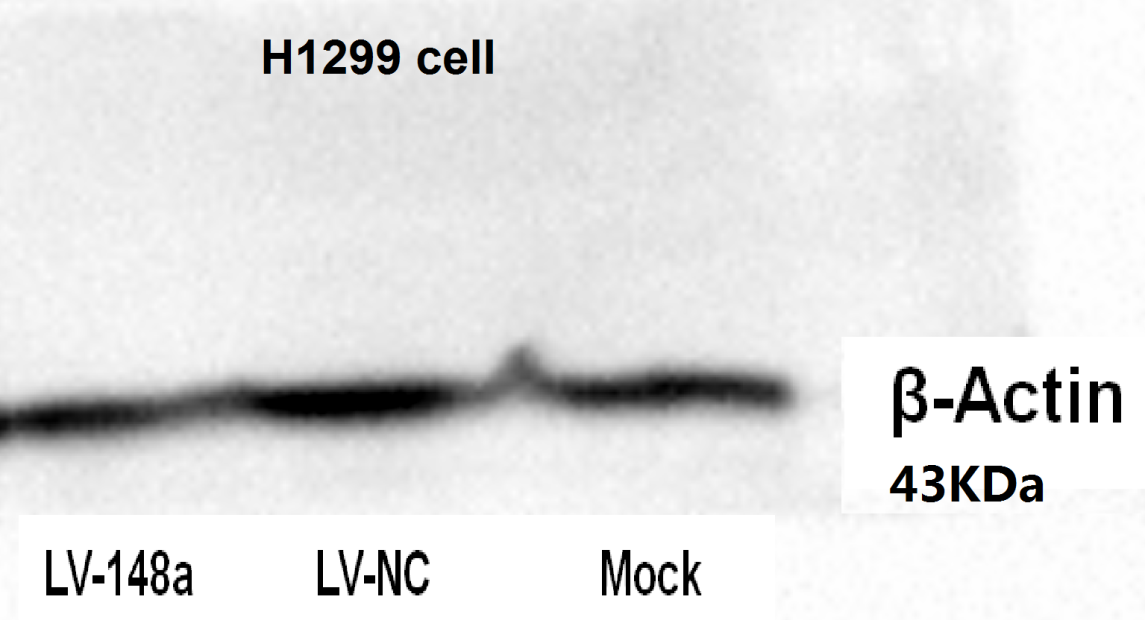

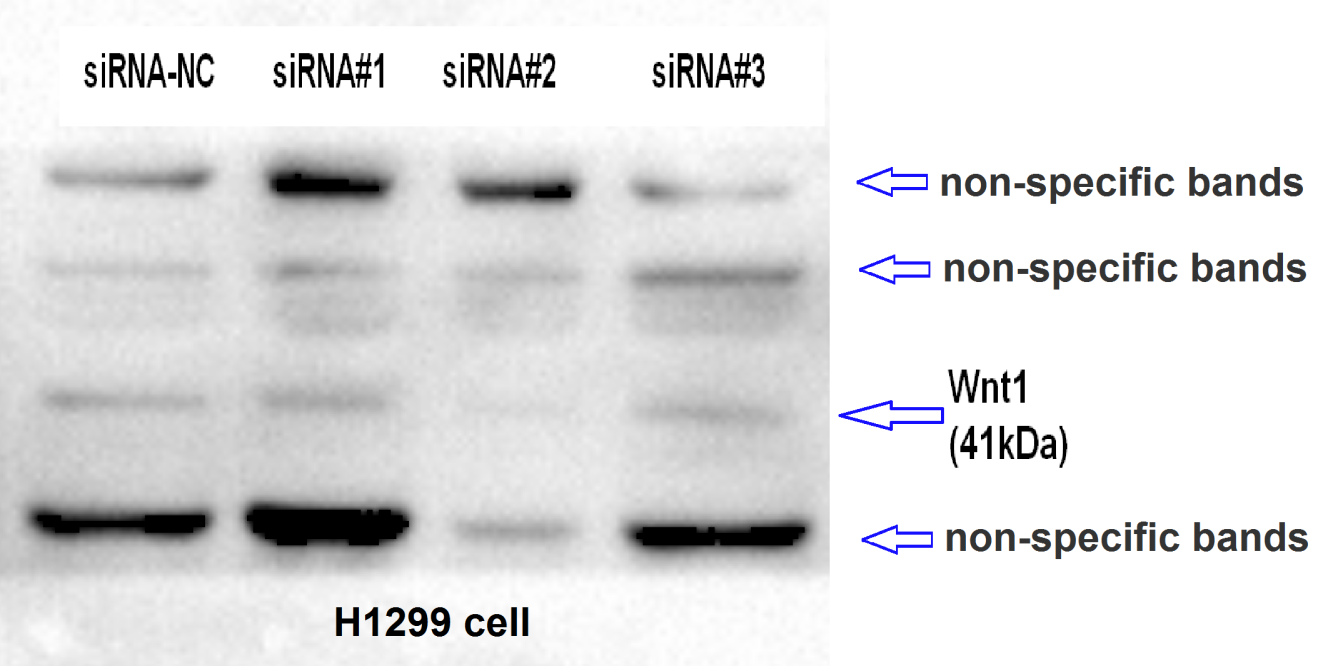

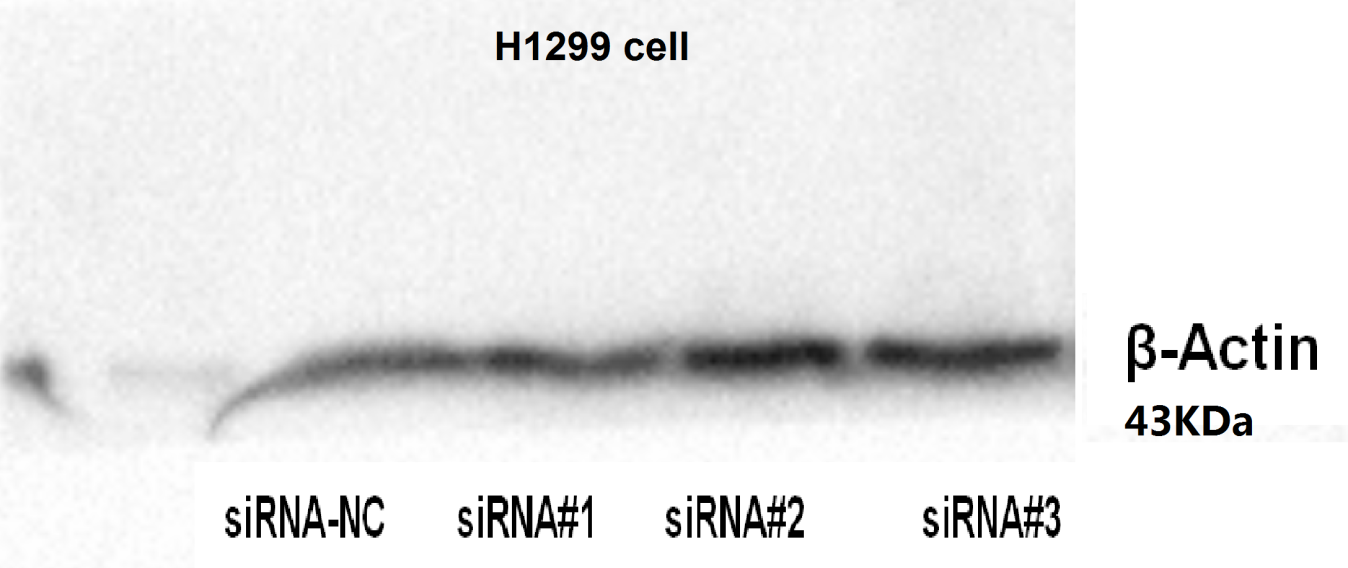

Supplement: S1 File — (DOCX) [file pone.0171751.s001.docx]
